# A Missense Variant in *TP53* Could Be a Genetic Biomarker Associated with Bone Tissue Alterations

**DOI:** 10.3390/ijms25031395

**Published:** 2024-01-23

**Authors:** Ricardo Usategui-Martín, Nadia Galindo-Cabello, Salvador Pastor-Idoate, José María Fernández-Gómez, Álvaro del Real, Diego Ferreño, Rebeca Lapresa, Francisco Martín-Rodriguez, José A. Riancho, Ángeles Almeida, José Luis Pérez-Castrillón

**Affiliations:** 1 Department of Cell Biology, Genetics, Histology and Pharmacology, Faculty of Medicine, University of Valladolid, 47003 Valladolid, Spain; nadiaregina.galindo@uva.es (N.G.-C.); josefg@uva.es (J.M.F.-G.); 2 IOBA—Eye Institute, University of Valladolid, 47011 Valladolid, Spain; spastori@ioba.med.uva.es; 3 Department of Medicine and Psychiatry, Faculty of Medicine, Valdecilla Research Institute (IDIVAL), University of Cantabria, 39011 Santander, Spain; alvarodel.real92@gmail.com (Á.d.R.); rianchoj@unican.es (J.A.R.); 4 Laboratory of the Materials Science and Engineering Division—LADICIM, Faculty of Civil Engineering, University of Cantabria, 39011 Santander, Spain; diego.ferreno@unican.es; 5 Institute of Functional Biology and Genomics, University of Salamanca, CSIC, 37008 Salamanca, Spain; rebecalrg@usal.es (R.L.); aaparra@usal.es (Á.A.); 6 Institute of Biomedical Research of Salamanca (IBSAL), University Hospital of Salamanca, University of Salamanca, CSIC, 37008 Salamanca, Spain; 7 Department of Medicine, Dermatology and Toxicology, Faculty of Medicine, University of Valladolid, 47003 Valladolid, Spain; francisco.martin.rodriguez@uva.es; 8 Internal Medicine Department, Marqués de Valdecilla University Hospital, 39008 Santander, Spain; 9 Internal Medicine Department, University Hospital Rio Hortega of Valladolid, 47012 Valladolid, Spain

**Keywords:** metabolic bone diseases, osteoporosis, *TP53*, p53, apoptosis and gene polymorphism

## Abstract

Metabolic bone diseases cover a broad spectrum of disorders that share alterations in bone metabolism that lead to a defective skeleton, which is associated with increasing morbidity, disability, and mortality. There is a close connection between the etiology of metabolic bone diseases and genetic factors, with *TP53* being one of the genes associated therewith. The single nucleotide polymorphism (SNP) Arg72Pro of *TP53* is a genetic factor associated with several pathologies, including cancer, stroke, and osteoporosis. Here, we aim to analyze the influence of the *TP53* Arg72Pro SNP on bone mass in humanized Tp53 Arg72Pro knock-in mice. This work reports on the influence of the *TP53* Arg72Pro polymorphism in bone microarchitecture, OPG expression, and apoptosis bone status. The results show that the proline variant of the TP53 Arg72Pro polymorphism (Pro72-p53) is associated with deteriorated bone tissue, lower OPG/RANK ratio, and lower apoptosis in bone tissue. In conclusion, the *TP53* Arg72Pro polymorphism modulates bone microarchitecture and may be a genetic biomarker that can be used to identify individuals with an increased risk of suffering metabolic bone alterations.

## 1. Introduction

Metabolic bone diseases cover a broad spectrum of disorders that share alterations in bone metabolism that lead to a defective skeleton [1]. The most prevalent metabolic bone disease worldwide is osteoporosis (OMIM: 166710), which is a chronic, progressive, systemic disease associated with reduced bone mineral density (BMD) and alterations in the microarchitecture of bone tissue [2,3]. The underlying mechanism associated with osteoporosis is an imbalance in bone formation and resorption which leads to a reduction in BMD and increased bone fragility [1,4]. Bone fragility fractures are the most relevant clinical complication of osteoporosis [5,6] and have become a public health issue that increases morbidity, disability, and mortality [7]. The identification of risk biomarkers for osteoporotic fracture is key to the establishment of early treatment and to avoid the appearance of the first fracture or re-fractures. Avoiding the first fracture is important to stop the cascade of subsequent fractures. In this sense, the use of genetic biomarkers could be a useful tool to identify individuals at higher risk.

The most accepted hypothesis of the etiopathogenic of osteoporosis and bone fragility fracture is the combined action of environmental and genetic factors. Many risk factors are involved, including age, physical activity, medication use, and coexisting diseases. But one of the most important is a positive family history, as it emphasizes the relevance of genetic predisposition in the pathogenesis of osteoporosis and bone fracture [8,9,10,11]. Several hundreds of genetic loci have been associated with osteoporosis, low BMD, and fragility fractures [12,13]. One of the genes so far associated therewith is *TP53* [14], the Arg72Pro missense variant which has been linked to an increased risk of osteoporosis. The proline allele has been associated more frequently with patients with osteoporosis, suggesting that the *TP53* p.Arg72Pro variant may be a risk factor for osteoporosis [15]. The TP53 gene encodes protein p53 with 393 amino acids. The principal role of p53 is to promote cell cycle arrest, apoptosis, and cell senescence [16]. The p53 protein is also involved in bone metabolism; it regulates osteoblast differentiation, bone formation, osteoblast-dependent osteoclast differentiation, and bone remodeling [17,18]. The Arg72Pro polymorphism results in a change in the structure of the p53 protein in a proline-rich domain. This genetic variant is located in a domain involved in the apoptotic role of p53 [19,20,21]. In this sense, it has been reported that the arginine allele of the Arg72Pro polymorphism is associated with more apoptosis induction than the proline variant [19,20,21]. The p. Arg72pro polymorphism in the p53 protein has been frequently associated with the pathophysiology of a variety of diseases including bone metabolism alterations. According to the results from Jia et al. [15], the *TP53* p. Arg72Pro missense variant could be a genetic biomarker of osteoporosis risk.

In such a scenario, and with an existing need to better understand the genetic factors determining osteoporosis and fragility fractures, this study aimed to obtain a deeper understanding of the pathophysiological bone consequences of the *TP53* Arg72Pro missense genetic variant using a humanized mouse model. The putative rol of the *TP53* p.Arg72Pro polymorphism in the susceptibility of suffering bone fragility or osteoporosis will be evaluated.

## 2. Results

**Figure 1:**
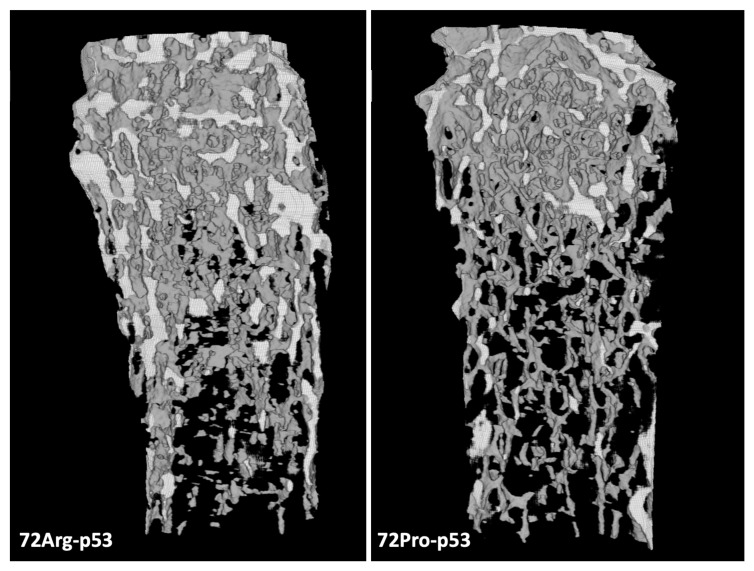
Micro-computed tomography (μCT) results for the femur trabecular area of 72Arg-p53 and 72Pro-p53 mice.

**Figure 2:**
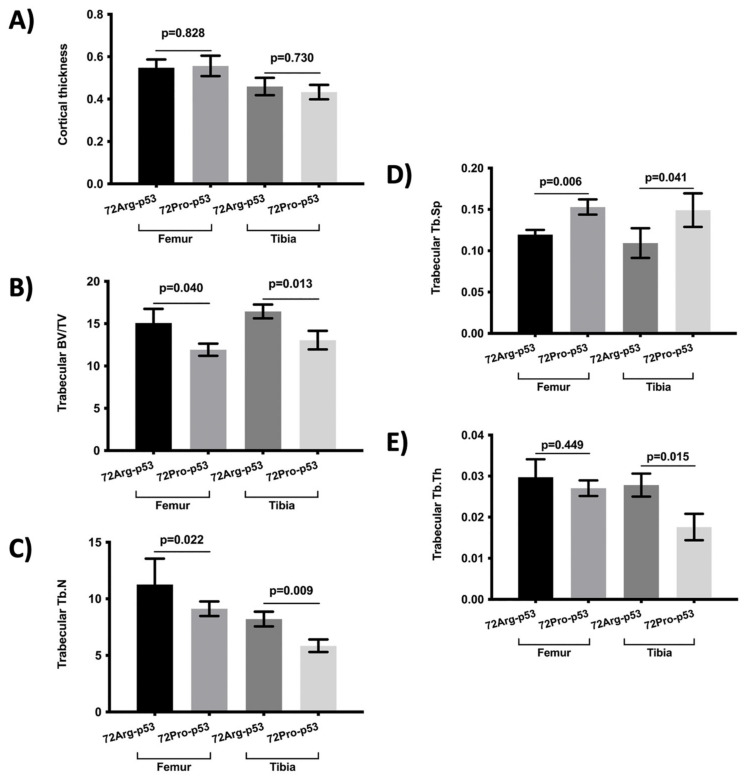
Comparison of bone morphometry parameters between 72Arg-p53 and 72Pro-p53 mice in the femur and tibia: (**A**) cortical thickness, (**B**) trabecular BV/TV, (**C**) Tb.N, (**D**) Tb.Sp, and (**E**) Tb.Th. Variables are presented as the mean (standard deviation). Bars represent the mean values and their respective standard deviation. BV/TV: percent bone volume; Tb.Th: trabecular thickness; Tb.Sp: trabecular separation and Tb.N: trabecular number.

The results from the micro-computed tomography (μCT) showed that the bone from 72Pro-p53 mice was more deteriorated than that of 72Arg-p53 mice. A representative comparison of femur trabecular areas between 72Arg- and 72Pro-p53 mice is shown in Figure 1, with deterioration shown in the trabecular area in the 72Pro-p53 mice. The bone histomorphometry parameters were evaluated in humanized 72Arg-p53 and 72Pro-p53 mice (Figure 2). The results showed that the 72Pro-p53 mice had a lower trabecular bone mass, both at the femur and the tibia, with the lower femur and tibia bone volume over total volume (BV/TV), femur and tibia trabecular separation (Tb.Sp), and tibia trabecular thickness (Tb.Th) included. In addition, the 72Pro-p53 mice had lower femur and tibia trabecular number (Tb.N) than the 72Arg-p53 mice (Figure 2). The analysis of the cortical thickness did not show statistical differences between both genotypes. These results are also shown in Supplementary Table S1.

**Figure 3:**
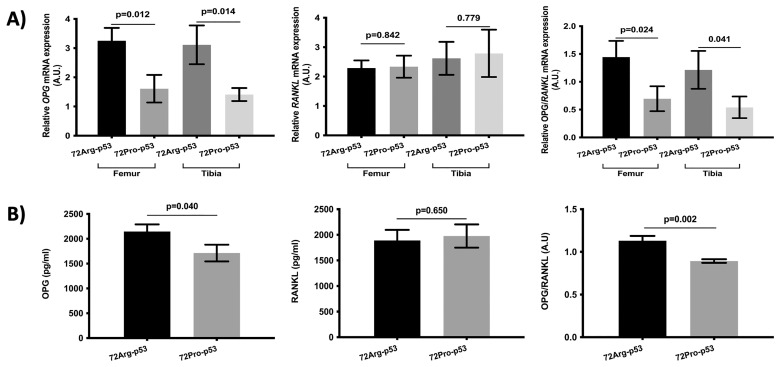
Comparison of the OPG and RANKL analyses between the 72Arg-p53 and 72Pro-p53 mice. Relative mRNA expression of the *OPG* gene and the *RANKL* gene and the *OPG/RANKL* ratio in the femur and tibia bone tissue (**A**). OPG and RANKL plasma protein levels and OPG/RANKL plasma ratio (**B**). Bars represent mean values and their respective standard deviation. A.U.: arbitrary units.

The results of the analysis of osteoclast maturation- and activation-related genes showed lower relative expression of osteoprotegerin (*OPG*) in the femur and tibia bone tissue in the 72Pro-p53 mice. Also, the *OPG/RANKL* ratio was lower in the femur and tibia of the 72Pro-p53 mice (Figure 3A). In the case of plasma, the OPG protein levels and OPG/RANKL plasma protein ratio were lower in the 72Pro-p53 mice than in the 72Arg-p53 mice (Figure 3B).

**Figure 4:**
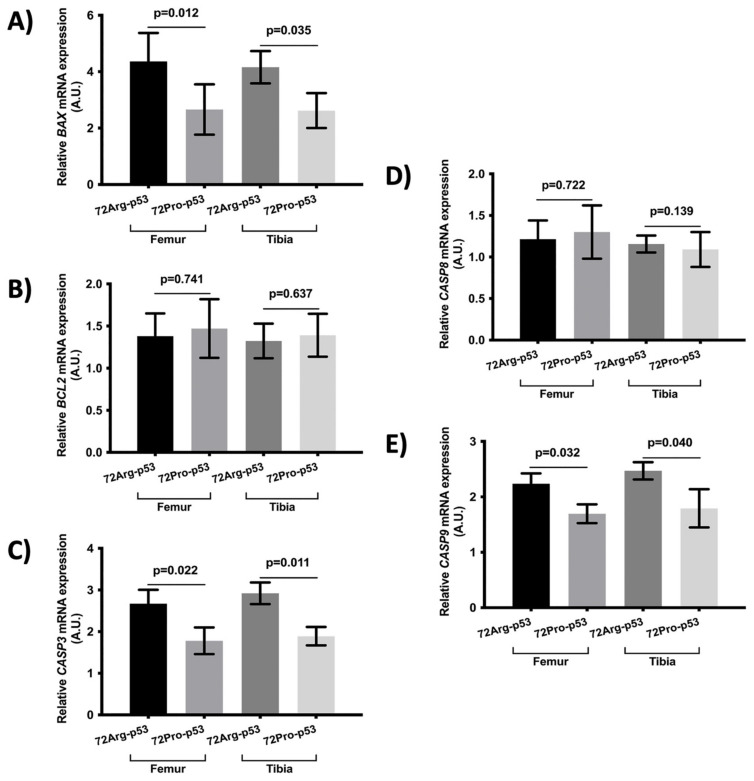
Relative mRNA expression of apoptosis-related genes in the femur and tibia bone tissue. Relative mRNA expression of the *BAX* gene (**A**), *BCL2* gene (**B**), *CASP3* gene (**C**), *CASP8* gene (**D**), and *CASP9* gene (**E**). Bars represent mean values and their respective standard deviation. A.U.: arbitrary units.

**Figure 5:**
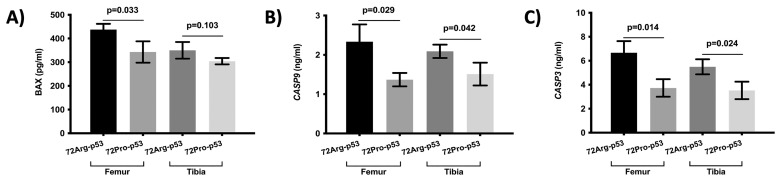
Expression of apoptosis-related proteins in the femur and tibia bone tissue. Protein expression of *BAX* (**A**), *CASP9* (**B**), and *CASP3* (**C**). Bars represent mean values and their respective standard deviation.

The results also showed statistical differences in the expression of genes involved in apoptosis between 72Arg-p53 and 72Pro-p53 mice. Relative mRNA quantification of gene expression involved in apoptosis showed lower relative expression of *BAX*, *CASP3*, and *CASP9* in the femur and tibia of the 72Pro-p53 mice. The expression of these genes was higher in the 72Arg-p53 mice (Figure 4). The relative expression of *BCL2* and *CASP8* did not show statistical differences between the 72Arg-p53 and 72Pro-p53 mice (Figure 4). In addition, the expression of apoptosis-related proteins in the femur and tibia bone tissue was also analyzed. The results showed that the protein expression of BAX, CASP3, and CASP9 was lower in the femur bone tissue of the 72Pro-p53 mice than that of the 72Arg-p53 mice. The expression of CASP3 and CASP9 proteins was also lower in the tibia bone tissue of the 72Pro-p53 mice (Figure 5). The relative expression of genes involved in inflammation and oxidative stress did not yield statistical differences (Supplementary Figures S1 and S2).

## 3. Discussion

Osteoporosis, characterized by low BMD and alteration in bone microarchitecture, is the most common metabolic bone disease [2,3]. Our results are in line with the concept that genetic predisposition could be crucial in its etiology. The results show that the proline variant of the *TP53* Arg72Pro polymorphism is associated with deteriorated bone tissue, reinforcing the hypothesis that the *TP53* gene could be involved in determining individual osteoporosis susceptibility [14,15]. These results indicate that this genetic variant could be a good indicator of osteoporosis risk. Morphologically, it has been associated with a deterioration in bone microarchitecture and there is a biological explanation for this: lower apoptosis and decreased osteoprotegerin activity. These data could imply that this higher-risk subgroup could benefit from treatment with bisphosphonates or denosumab, drugs that inhibit proliferation and increase osteoclast apoptosis.

Protein p53 is a tumor suppressor, that is, a stress sensor that induces apoptosis, cell cycle arrest, or senescence [16]. It has also been published that p53 plays a key role in bone metabolism [17,18]. Protein p53 could be involved in osteogenesis [22]; it modulates osteoblastic and osteoclastic differentiation, increasing the Notch signaling pathway [17,23]. P53 has also been associated with the regulation of OPG synthesis; it has been reported that there is a negative correlation between *TP53* gene modifications and OPG regulation [24]. OPG is a soluble member of the tumor necrosis factor receptor superfamily. It is a decoy receptor for RANKL because OPG inhibits osteoclastic bone resorption by interfering with the binding of RANKL to RANK [25,26]. Multiple single nucleotide polymorphisms (SNPs) have been identified in *TP53* [27,28]; one of the most studied is the Arg72Pro variant. This genetic polymorphism has been associated with various cancers, inflammatory diseases, and stroke [29,30,31,32]. Also, the *TP53* Arg72Pro genetic variant has been found to be involved in osteoporosis; the proline variant has been associated with an increased risk of suffering osteoporosis [15]. In this sense, our results showed that the proline allele of the *TP53* Arg72Pro polymorphism is associated with a higher level of deterioration of bone tissue in the trabecular femur and tibia. In addition, we report that the proline allele has been found to be associated with lower *OPG* gene expression and a lower *s/RANKL* ratio in the femur and tibia, lower OPG protein plasma levels, and a lower OPG/RANKL plasma protein ratio. The crucial role of protein p53 in the regulation of OPG status [24] and therefore bone metabolism has been reported [18]. Our results support the hypothesis that the *TP53* Arg72Pro variant could be associated with negative regulation of OPG and, therefore, increased osteoclastogenesis and, consequently, a more impaired bone microarchitecture.

Also, P53 has a crucial role in apoptosis induction. Arg72Pro SNP is in a proline-rich domain involved in the apoptotic role of p53 [19,20,21]. In this sense, it has been reported that the arginine allele of the Arg72Pro polymorphism is associated with more apoptosis induction than the proline variant [19,20,21]. Our report showed that the arginine variant could be associated with more apoptosis induction in bone tissue, specifically with the intrinsic apoptotic pathway. The results associated the arginine variant of the Arg72Pro *TP53* polymorphism with altered expression of the *BAX*, *CASP3*, and *CASP9* genes and proteins in the bone tissue. Although genetic factors associated with inflammation and oxidative stress have been associated with bone osteoporotic fracture [33,34], our results did not show modifications in inflammation and oxidative stress gene expression in bone tissue with regard to the *TP53* Arg72Pro polymorphism. Many risk factors have been associated with alterations in bone metabolism and the risk of suffering bone metabolism diseases [6]. Our hypothesis is that the metabolic bone response to these risk factors could be different depending on the *TP53* Arg72Pro genetic variant. The results of this work indicate that the proline allele of the *TP53* Arg72Pro polymorphism is associated with decreased apoptotic function, lower OPG/RANKL ratio, and worsened bone microarchitecture. It could be speculated that reduced levels of apoptosis may be followed by a more aggressive cellular response and thus more bone alterations. On the other hand, and in addition, the proline allele of *TP53* Arg72Pro SNP has been associated with increased activation of the NF-kB pathway [35], which is crucial in osteoclastogenesis [36,37]. Also, p53 is involved in the regulation of OPG [24] which has a crucial role in osteoclastic bone resorption [25,26]. In this sense, we report an association between the proline allele of the *TP53* Arg72Pro variant and *OPG* expression; the proline variant was associated with lower gene expression and lower OPG/RANKL ratio. Hence, the proline variant of the *TP53* Arg72Pro polymorphism could be associated with more osteoclast maturation and activation and therefore more bone resorption. This mutation may have special significance in the elderly population. The decrease in apoptosis increases the percentage of senescent cells in bone tissue, which are viable cells but with an irreversible arrest of the cell cycle. The p53/21 metabolic pathway plays a role in this arrest. Clinical–functional repercussions increase morbidity and mortality among this population [38].

The main limitation of the study is that we did not evaluate the influence of the *TP53* Arg72Pro genetic variant in bone metabolism under conditions of bone injury and that we only analyzed bone quality using μCT. Another limitation could be the lack of in vitro studies to analyze the consequence of the mutation, but we consider that this is not necessary since we observed the effect of the mutation in an in vivo model. On the other hand, and as a main strength, this work summarizes, for the first time, the influence of the *TP53* Arg72Pro polymorphism in *OPG* gene expression, OPG/RANKL ratio, bone microarchitecture, and apoptosis bone status.

In conclusion, we described the influence of the *TP53* Arg72Pro polymorphism in bone microarchitecture, reinforcing the hypothesis that the *TP53* Arg72Pro genetic variant could be crucial in osteoporosis risk. The *TP53* Arg72Pro missense variant could be a genetic biomarker to identify individuals with an increased risk of suffering osteoporosis.

## 4. Materials and Methods

### 4.1. Animals

Humanized *TP53* Arg72Pro knock-in (KI) mice were used. No differences in whole skeleton, body weight, and survival between the two genotypes were described. The animals were bred at the Animal Welfare and Research Service of the University of Valladolid in accordance with Spanish law (RD 53/2013). All experiments were carried out in compliance with the applicable international rules and policies—European Union Directive for Protection of Vertebrates Used for Experimental and Other Scientific Ends (2010/63/EU)—and were reviewed and approved by the University of Valladolid Institutional Committee for Animal Care and Use.

### 4.2. Micro-Computed Tomography (μCT)

The bone histomorphometry was analyzed using μCT. The femur and tibia specimens were scanned using a high-energy micro-computed tomography system 527 (SkyScan 11732, Bruker Micro-CT, Kontich, Belgium) and Sky-scan 1172 µCT data acquisition software. Since the aim was to maximize the resolution of the samples, the pixel size was reduced to the minimum, with it reaching a pixel size of 6.7 µm and a voxel size of 300.76 µm^3^. Scanning was performed at 50 kV and an Al 0.5 mm filter was used to reduce noise during scanning. During the reconstruction, parameters were used to correct possible beam hardening, ring artifacts, and misalignment problems. Maximum and minimum values for the attenuation coefficient were established. The minimum value was set at 0. For the maximum value, the critical section of all scans, the one with the maximum attenuation coefficient value, was selected at the operator’s discretion. Once this section was defined, the maximum value within the histogram was determined and a margin of error of 10% was applied. Finally, the cortical and trabecular areas of the tibia and femur were analyzed. The trabecular bone analysis was performed on the distal femur and proximal tibia areas. The regions of interest covered a total of 3 mm, specifically 2 mm below the growth plate. For the analysis of the cortical area, 3 mm of the central regions of the femur and tibia were selected at a distance of 15 mm from the growth plate of the tibia and femur. The regions of interest for delineation in each image were fully automated and assessed, as described by Bruker’s instructions [39,40]. The scan parameters have been included in Supplementary Table S2.

The structural parameter of the cortical bone analyzed was cortical thickness. In the trabecular bone, bone volume over total volume (BV/TV), trabecular number (Tb.N), trabecular thickness (Tb.Th), and trabecular separation (Tb.Sp) were analyzed. The BV/TV parameter indicates the ratio of bone tissue within the whole sample, and Tb.N, Tb.Th, and Tb.Sp determine the quality of the trabecular bone.

### 4.3. Sample Processing and RNA Extraction

Total RNA was extracted from bone tissue using the RNA easy Mini Kit (QIAGEN, Barcelona, Spain), following the manufacturer’s instructions. Bone tissue was submerged in RNA-stabilizing solution (RNAlater, Invitrogen, Waltham, MA, USA) and stored at −80 °C until RNA extraction. Bone tissue was homogenized with polytron tissue homogenizer using Trizol reagent (Invitrogen). Finally, RNA was extracted using a phenol–chloroform mixture, precipitated in ethanol, and purified using RNase-free columns. RNA quantity and purity were determined according to absorbance in a spectrophotometer (NanoDrop 2000, Thermo, Waltham, MA, USA).

### 4.4. Reverse Transcription and Real-Time Quantitative PCR

Complementary DNA (cDNA) was synthesized by means of reverse transcription using a High-Capacity cDNA Reverse Transcription Kit (Applied Biosystems, Foster City, CA, USA). The relative quantitative real-time polymerase chain reaction (qPCR) was performed using SYBR Green PCR master mix (Applied Biosystems) and mice-specific primer sets (Supplementary Table S3). Relative mRNA expression was analyzed for apoptosis, inflammation, and oxidative stress-related genes. The qPCR experiments were conducted using the Applied Biosystems 7500 Real-Time PCR System under the following conditions: 95 °C, 10 min; 40 cycles of 95 °C, 15 s; 60 °C, 1 min; and a final melting curve step. The *GAPDH* gene was used as a housekeeping gene for the normalization of the expression level of mRNA. The threshold cycle was determined for each reaction, and gene expression was quantified using the 2−ΔΔCt method [41]. All qPCR reactions were performed in triplicate.

### 4.5. Enzyme-Linked Immunosorbent Assay

The plasma levels of OGP and RANKL proteins (involved in bone metabolism) were measured using the enzyme-linked immunosorbent assay (ELISA) method, following the instructions of the manufacturer. Bone tissue levels of BAX, CASP3, and CASP9 proteins (involved in apoptosis) were also measured. The samples were measured in triplicate. Absorbance was determined using a spectrophotometer ELx800 Universal Microplate Reader (Bio-Tek Instruments Inc., Winooski, VT, USA) at 450 nm with a wavelength correction of 620 nm.

### 4.6. Statistical Analysis

Continuous variables were expressed as the mean (standard deviation). The Kolmogorov–Smirnov test was used to analyze the distribution of continuous variables. As for normally distributed variables, the analysis of variance t-test was applied. In the case of non-normally distributed variables, the groups were compared using the Mann–Whitney U-test (two groups) or the Kruskal–Wallis test (more than two groups). A *p*-value < 0.05 was considered significant. All analyses were performed using the SPSS version 22.0 statistical package (SPSS, Chicago, IL, USA).

## Data Availability

All of the results are in the main manuscript.

## Supplementary Materials

The following supporting information can be downloaded at: https://www.mdpi.com/article/10.3390/ijms25031395/s1.
