# Supplementary material for: A Missense Variant in TP53 Could Be a Genetic Biomarker Associated with Bone Tissue Alterations
Source: Int J Mol Sci. 2024 Jan 23;25(3):1395. doi: 10.3390/ijms25031395 (PMC10855390; doi:10.3390/ijms25031395)
Supplement: Supplementary file 1 [file ijms-25-01395-s001.zip › ijms-2809986-supplementary.pdf]

A Missense Variant in TP53 Could Be a Genetic Biomarker Associated to Bone Tissue Alterations

Supplementary Materials

Supplementary Table S1. Bone morphometry parameters in 72Arg-p53 and 72Pro-p53 mice.

Bone morphometry parameters (mean (SD))	Femur			Tibia		
	72Arg-p53	72Pro-p53	p-value	72Arg-p53	72Pro-p53	p-value
**Cortical Thickness**	0.5477 (0.039)	0.5561 (0.0481)	0.808	0.4593 (0.041)	0.4329 (0.034)	0.730
**Trabecular percent bone volume (BV/TV)**	15.1 (1.6)	11.9 (0.7)	0.040	16.4 (0.8)	13.1 (1.1)	0.013
**Trabecular number (Tb.N)**	11.2 (2.3)	9.1 (0.6)	0.022	8.2 (0.6)	5.9 (0.4)	0.009
**Trabecular separation (Tb.S)**	0.12 (0.005)	0.15 (0.009)	0.006	0.10 (0.018)	0.14 (0.020)	0.041
**Trabecular thickness (Tb.Th)**	0.029 (0.004)	0.027 (0.001)	0.449	0.027 (0.002)	0.017 (0.003)	0.015

Supplementary Table S2. Scan parameters.

**Variable**	**Standard Unit**	**Value**
Voxel size	µm3	300.76
Source Voltage	kV	50
Source Current	µA	100
Exposure time	ms	4920
Frame averaging	N	5
Projections	N	130

Supplementary Table S3. Gene-specific primer sequences for real-time quantitative PCR.

**Gene**	**Primer sequence (5´to 3`)**
OPG-Fwd	TACCTGGAGATCGAATTCTGCTT
OPG-Rev	CCATCTGGACATTTTTTGCAAA
RANKL-Fwd	CCTGAGGCCCAGCCATTT
RANKL-Rev	CTTGGCCCAGCCTCGAT
BCL2-Fwd	TTCAGGGATGGGGTGAACTG
BCL2-Rev	CACAGGGCGATGTTGT
BAX-Fwd	CCCGAGAGGTCTTCTTCC
BAX-Rev	GCCTTGAGCACCAGTTTG
CASP3-Fwd	GTGGACTCTGGGATCTATCT
CASP3-Rev	CCATGAATGTCTCTCTGAGG
CASP8-Fwd	CGGGAAGACATAACCCAACT
CASP8-Rev	GTGGGATAGGATACAGCAGA
CASP9-Fwd	AGTTCCCGGGTGCTGTCTAT
CASP9-Rev	GCCATGGTCTTTCTGCTCAC
IL1-Fwd	ACAAACCACCCGTTTCACCT
IL1-Rev	ATGGGTGGAGGGTTCACTCT
IL6-Fwd	GAGGATACCACTCCCAACAGACC
IL6-Rev	AAGTGCATCATCGTTGTTCATACA
IL10-Fwd	GCTCTTACTGACTGGCATGAG
IL10-Rev	CGCAGCTCTAGGAGCATGTG
TNFa-Fwd	ACCCTCACACTCAGATCATCTTC
TNFa-Rev	TGGTGGTTTGCTACGACGT
COX2-Fwd	AACCGAGTCGTTCTGCCAAT
COX2-Rev	GGGACTGCTCATGAGTGGAG
CYBB-Fwd	GACCTCCCAGAGAACACAGC
CYBB-Rev	ACCCTAGCCTGCTTATGGGA
GPX6-Fwd	GTCACGGTTTTGGGCTTTCC
GPX6-Rev	CCCCCTTCTCAAAGAGCTGG
SOD1-Fwd	GGAACCATCCACTTCGAGCA
SOD1-Rev	CCCATGCTGGCCTTCAGTTA
TXN2-Fwd	CTGTAGCCTGACCGCACTAG
TXN2-Rev	AGGCAGGAGAGGAAATGTGC
TXNRD1-Fwd	CTCGGAGGAACGTGTGTGAA
TXNRD1-Rev	AGTTGCGCGAGTCTTTCAGA
GAPDH-Fwd	TGCACCACCAACTGCTTAGC
GAPDH-Rev	CACCACCTTCTTGATGTCATCA

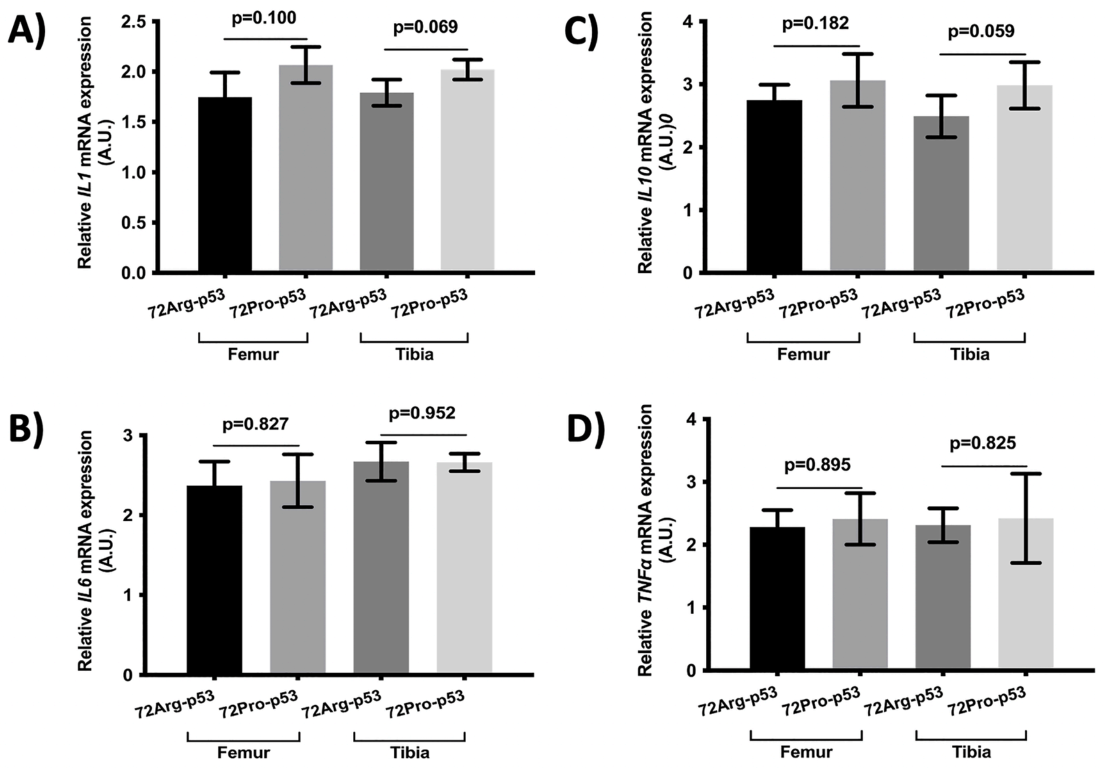

Supplementary Figure S1. Relative mRNA expressions of inflammation-related genes in femur and tibia bone tissue. Relative mRNA expressions of IL1 gene (**A**), IL6 gene (**B**), IL10 gene (**C**), and TNFa gene (**D**). Bars represent mean values and their respective standard deviation. A.U.: arbitrary units

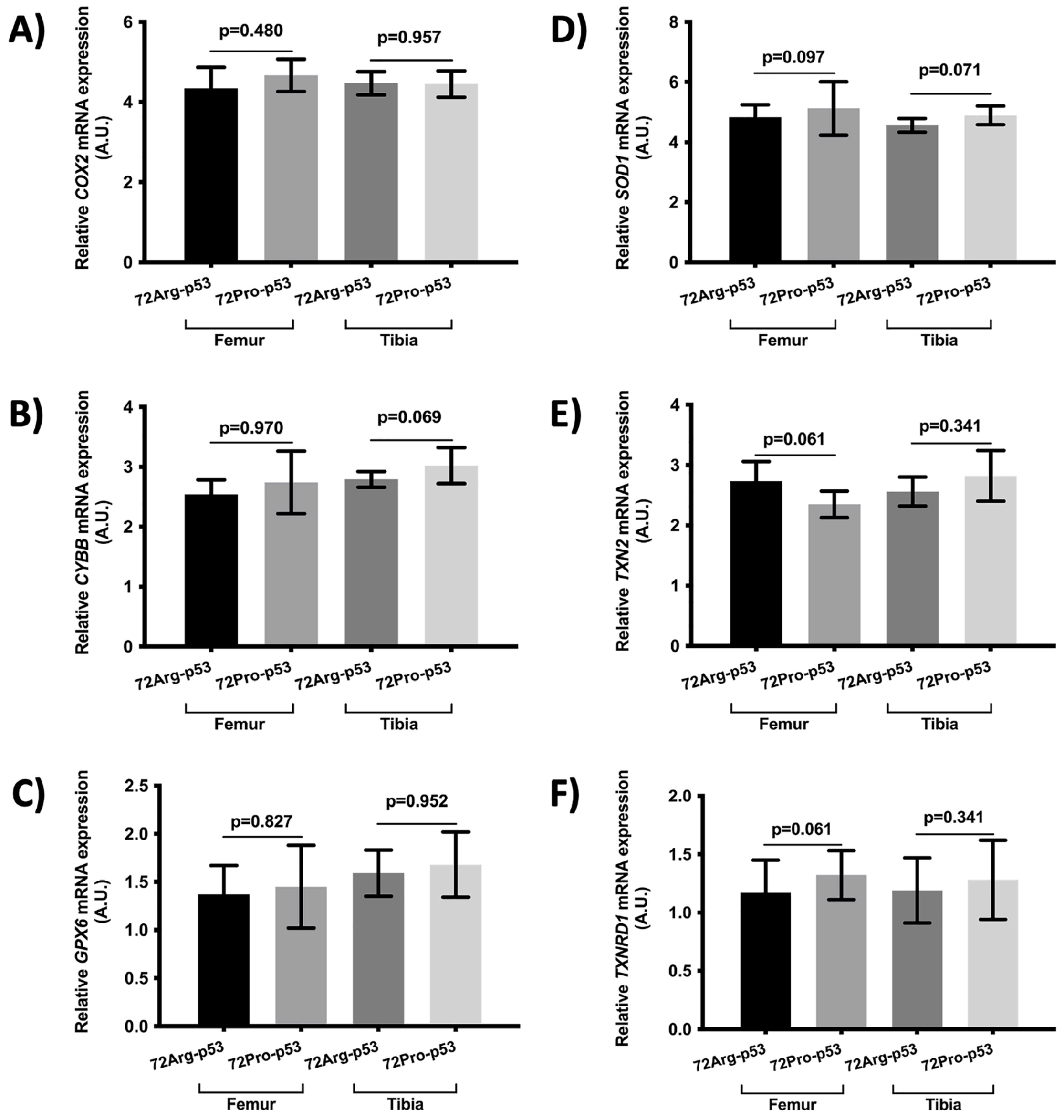

Supplementary Figure S2. Relative mRNA expressions of oxidative stress-related genes in femur and tibia bone tissue. Relative mRNA expressions of COX2 gene (**A**), CYBB gene (**B**), GPX6 gene (**C**), SOD1 gene (**D**), TXN2 gene (**E**), and TXNRD1 gene (**F**). Bars represent mean values and their respective standard deviation. A.U.: arbitrary units.
